# Acid-Neutralizing Omeprazole Formulation for Rapid Release and Absorption

**DOI:** 10.3390/pharmaceutics17020161

**Published:** 2025-01-25

**Authors:** Sreela Ramesh, Vít Zvoníček, Daniel Pěček, Markéta Pišlová, Josef Beránek, Jiří Hofmann, Aleksandra Dumicic

**Affiliations:** Zentiva, k.s., U Kabelovny 130, 10237 Prague, Czech Republic; sreela.ramesh@zentiva.com (S.R.); vit.zvonicek@zentiva.com (V.Z.); marketa.pislova@zentiva.com (M.P.); jiri.hofmann@zentiva.com (J.H.)

**Keywords:** omeprazole, neutralization, absorption, pharmacokinetics, stability, drug release

## Abstract

**Background/Objectives**: Omeprazole undergoes degradation in acidic conditions, which makes it unstable in low pHs found in the gastric environment. The vast majority of already marketed omeprazole formulations use enteric polymer coatings to protect the drug from exposure to acidic pH in the stomach, allowing for drug release in the small intestine where the pH is higher. This study aimed to explore the technical aspects of using stomach acid neutralizers as an alternative to polymeric coatings for omeprazole. **Methods**: After evaluating various neutralizers, magnesium oxide and sodium bicarbonate were chosen to be incorporated into capsules containing omeprazole, which then underwent in vitro dissolution testing to assess their ability to maintain optimal pH levels and ensure appropriate dissolution kinetics. Hygroscopicity and chemical stability of the selected formulation were tested to prove pharmaceutical quality of the product. An in vivo pharmacokinetic study was conducted to demonstrate the efficacy of the omeprazole–sodium bicarbonate formulation in providing faster absorption in humans. **Results**: Sodium bicarbonate was selected as the most suitable antacid for ensuring omeprazole stabilization. Its quantity was optimized to effectively neutralize stomach acid, facilitating the rapid release and absorption of omeprazole. In vitro studies demonstrated the ability of the formulation to neutralize gastric acid within five minutes. In vivo studies indicated that maximum concentrations of omeprazole were achieved within half an hour. The product met the requirements of pharmaceutical quality. **Conclusions**: An easily manufacturable, fast-absorbing oral formulation was developed as an alternative to enteric-coated omeprazole.

## 1. Introduction

Gastroesophageal reflux disease (GERD), as defined by the American College of Gastroenterology (ACG), is a condition in which the reflux of gastric contents into the esophagus results in a number of symptoms and complications [1]. GERD affects 10% to 20% of the western population, whereas in Asia, its presence is lower, affecting less than 5% of the people [2]. The disease is characterized in most people by repeated reflux of contents of the stomach leading to unpleasant heartburn, regurgitation, and several other problems [3]. It is caused by increased relaxation of the lower esophageal sphincter, which leads to regurgitation of gastric acid. If prolonged, the acid can cause damage to the tissues of gastroesophageal mucosa [4].

Acid-neutralizing agents, which work by neutralizing the acidic pH, are well-known to provide efficient support in the treatment of stomach hyperacidity. Nowadays, upon availability of various medications, they are mostly used to enable rapid relief [5]. Salts of calcium, magnesium, and sodium are some examples of commonly used antacids. While they achieve a high rate of neutralization, with short time needed to achieve marginally acidic to neutral pH in the stomach, sustained relief still stays unaddressed, as the relief obtained via acid neutralization diminishes with continued secretion of stomach acid [6].

Consequently, since the approval of omeprazole in 1989, proton pump inhibitors (PPIs) have become a widely spread choice of treatment for GERD and other acid-related disorders. They have proven to be better than earlier treatments in terms of gastric acid reduction [7]. They work by inhibiting the gastric H^+^/K^+^-ATPase via covalent binding to cysteine residues of the proton pump, thereby suppressing acid secretion in the stomach. For example, the inhibitory effect of omeprazole occurs within 1 h of administration, the maximum effect occurring in 2 h, reaching a plateau on the fourth day of the therapy [8].

Several issues associated with formulations of PPIs remain unresolved even after decades of use. The vast majority of available PPI formulations are based on enteric coating of the drug particles, which is a technologically complex and lengthy process. Such coatings also move drug release from stomach to higher pHs present in the small intestine. Even when the coating is dissolved, absorption of PPIs is still influenced by residues of enteric coating [9], potentially increasing variability of drug absorption. Thus, delayed release of PPIs is a consequence of acid-resistant coating of either drug-containing particles, or the whole tablets. A necessity for such acid-resistance, enteric coating originates from chemical instability of PPIs at low pHs [10]. Protection of the active pharmaceutical ingredient (API) is mainly achieved by synthetic polymers, e.g., cellulose acetate phthalate and methacrylic acid copolymers [11], which create a barrier against the acidic stomach environment. Enteric coatings, being insoluble in the stomach at acidic pHs, allow for drug release in the small intestine due to the increased pH [11].

**Figure 1:**
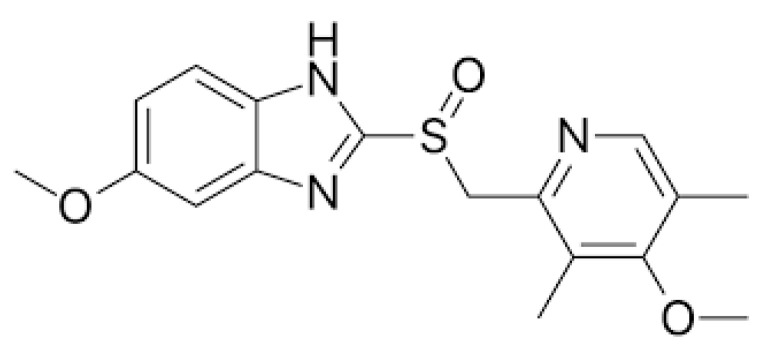
Molecular structure of omeprazole.

Omeprazole (Figure 1) is a commonly used PPI for the treatment of GERD and other conditions caused by excessive gastric acid [12]. Being a weak base, omeprazole acts as a prodrug that accumulates within the acid space of the parietal cell, where it is transformed via a proton-catalysed process to generate a sulphenamide, or corresponding unstable sulfenic acid. The sulphenamide (sulfenic acid) interacts covalently with the sulphydryl groups of cysteine residues in the extracellular domain of the H^+^K^+^-ATPase, thereby inhibiting its activity [13,14].

Omeprazole is a racemate and exists in two, S and R, optical isomeric configurations. The S-isomer generates higher plasma levels than the R-isomer, due to lower metabolic conversion by hepatic cytochromes (CYP2C19). The active molecular species (the sulphenamide) is non-chiral and is equipotent after generation from both isomers. The two isomers showed identical dose–response curves when tested in vitro for the inhibition of acid production in isolated gastric glands [13].

**Figure 2:**
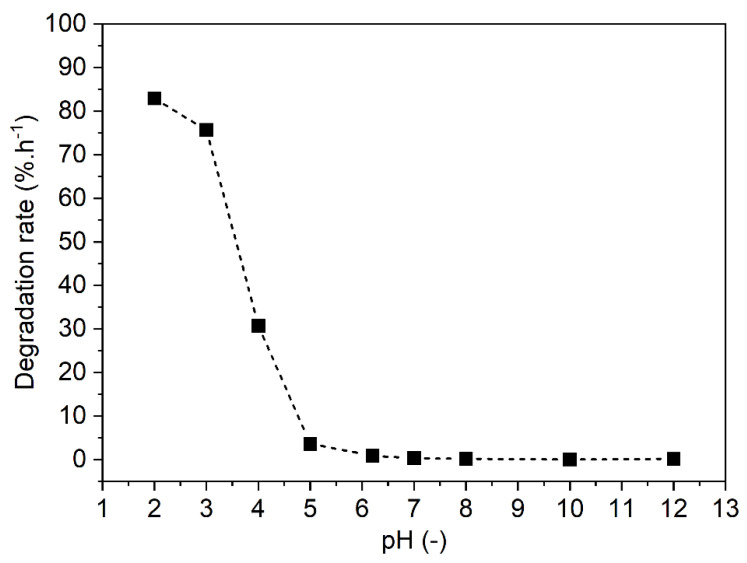
pH-dependent stability of omeprazole.

Omeprazole is a BCS class II lipophilic drug known for its acid lability, with stability increasing at higher pHs. As evident from the results of an in-house experimental evaluation (Section 2.2), the drug decomposes if directly exposed to the natural acidic pH of the stomach (Figure 2). The degradation half-life of omeprazole is reported to be 10 min at pH < 5, but 18 h at pH 6.5 [15]. Mass spectrometry analysis revealed that upon exposure to acidic conditions, omeprazole breaks down into five degradation products through mechanisms not limited to, but including, radical cation formation and nucleophilic attack of the pyridine ring on the imidazole ring [16]. If omeprazole degradation happens prematurely in the stomach, its bioavailability is significantly reduced. Thus, omeprazole is usually protected using an enteric coating [17].

Studies comparing several brands of omeprazole have reported that the acid-suppressive effect of those products markedly differed due to differences in the performance of the enteric coatings [18,19]. Even though enteric coatings provide chemical protection of omeprazole, challenges of the coating process itself significantly impact product performance, requiring a high level of technical knowledge and manufacturing experience.

There is, hence, a need for a formulation of omeprazole, which in addition to preventing its chemical degradation, will also allow for a simpler manufacturing process, immediate release of the drug, and its rapid absorption. Due to their acid-neutralizing effect, antacids can potentially be used to stabilize PPIs in the formulation. Zegerid (Salix Pharmaceuticals) is an example of such a formulation with an acid-neutralizing agent used along with omeprazole to prevent its decomposition by stomach acids. A clinical study showed that the Zegerid immediate-release formulation was efficient in providing a rapid onset of action, thereby helping to relieve heartburn associated with GERD [20,21]. This study, while proving fast relief originating from the administration of Zegerid, also showed overall non-inferiority to delayed-release Losec, which contains enteric-coated pellets [21]. An immediate-release formulation of esomeprazole, similarly, using sodium bicarbonate as a stabilizing agent, showed rapid and sustained suppression of gastric acidity [22].

While the mechanism of action of an immediate release formulation involving antacids is known, the reasoning behind selection of the most suitable antacid, from chemical and technological perspectives, still needs to be addressed. This study employs a technical approach to antacid selection, identifying the most effective antacid to be used in the formulation along with omeprazole. The aim of this research was to design an acid-neutralizing, stable, immediate-release formulation of omeprazole providing rapid release and absorption while employing standardly utilized manufacturing processes. An additional target of the formulation design was the size of the dosage form, i.e., the capsule as small as possible to aid easy swallowing. Chemical stability and hygroscopicity of the chosen formulation, indicating achieved pharmaceutical quality of the product, were tested in accordance with standard guidelines [23]. An in vivo pharmacokinetic study in humans was used to compare the product performance to acid-resistant, delayed-release Losec (omeprazole marketed in 1989 in the form of enteric-coated pellets filled in hard gelatin capsules).

## 2. Materials and Methods

All used materials were of pharmaceutical grade, satisfying requirements of PhEur. Losec 20 mg hard gastro-resistant capsules (Astra Zeneca AB, Stockholm, Sweden) were purchased from Austria. Sodium bicarbonate, other excipients, and solvents were of standard pharmaceutical quality and obtained from various commercial suppliers. Omeprazole for preparation of formulation was obtained from Uqiufa, Barcelona, Spain. Hard gelatin capsules were single sourced for the whole study, obtained from Qualicaps, Madrid, Spain, and were kept under standard storage conditions.

### 2.1. Preparation of Capsules

All capsules were prepared using standard manufacturing operations, consisting of direct mixing of omeprazole, antacid, and other excipients. The homogenized blends were filled in capsules of a suitable size. The finally selected formulation, filled in targeted capsule size ‘0’, was encapsulated using standard encapsulation equipment.

### 2.2. pH-Dependent Stability of Omeprazole

The degradation kinetics of omeprazole solutions of a concentration 1 mg/mL were measured individually at 40 °C in 10 mmol phosphate buffers at pHs 2, 3, 6.2, and 7.8, 10 mmol borate buffer at pH 10, and 10 mmol formate buffer at pHs 4 and 5. An Acquity UPLC BEH C8 100 × 2.1 mm column (Waters Corporation, Milford, MA, USA) was used for the HPLC analysis of omeprazole. Detection was performed by an in-built UV detector at 300 nm. The sampling rate of 10 points per second, flow rate of 0.3 mL/min, and an injection volume of 1.0 μL were used for the analyses. The column temperature was set to 30 °C. The degradation rate versus pH was used to construct the degradation graph.

### 2.3. Neutralizing Activity of Antacids

The neutralizing activity of antacids was measured by observing the pH change in 54 mL of 0.1 M HCl. A molar equivalent of each antacid was added to 5.4 mmol HCl in different beakers simultaneously and the suspensions were mixed at room temperature at power 15 using IKA^®^WERKE stirrers (IKA^®^-Werke GmbH & Co. KG, Staufen, Germany). The pHs of the suspensions were recorded using pH probes (Mettler Toledo, Columbus, OH, USA) and analyzed using online pH monitoring (Merel Instruments, Kamnica, Slovenia).

### 2.4. In Vitro Dissolution Experiment

The dissolution rate was measured using a USP 2 (Paddle, Agilent 708-DS by Agilent Technologies, Inc., Santa Clara, CA, USA) dissolution apparatus with online pH measuring probe. The measurements were performed using 900 mL of 6 mM HCl solution with a starting pH of 2.3 at 75 rpm, kept at 37 °C in a water bath. The capsules (omeprazole–NaHCO_3_ and omeprazole–MgO capsules) were analyzed without the sinkers. The concentration of omeprazole in the solution was recorded using a UV spectrophotometer (Analytik Jena GmbH+Co. KG, Jena, Germany) at 301 nm and 5 mm optical path length. The concentration was measured every 5 min up to 30 min, and then at 45, 50, and 60 min. All measurements were performed in triplicate.

### 2.5. Dynamic Vapor Sorption

Gravimetric moisture sorption analysis was carried out using a humidity- and temperature-controlled microbalance: DVS apparatus, DVS Advantage 1 (Surface Measurement Systems, London, UK), with a Cahn D200 recording ultra-microbalance with a mass resolution of ±0.1 μg. Prior to the start of the measurement, the omeprazole–NaHCO_3_ sample was dried at 0% RH under a nitrogen stream at 25 °C. Moisture uptake (reported relative to the dry weight) was monitored over a sorption/desorption range of 0–90% RH in increments of 10%.

### 2.6. Chemical Stability

Chemical stability of the capsules was tested in real-time according to the International Council for Harmonization of Technical Requirements for Pharmaceuticals for Human Use (ICH) guidelines [23]. The capsules, packed in PVDC/Al blisters, were stored at a temperature of 30 °C and a relative humidity of 65%. The methods described in the current PhEur monographs were used to test the stability of the capsules [24]. Assays of omeprazole, antacid, and the amount of impurities originating from omeprazole degradation were analyzed at regular time points (3, 6, 9, and 12 months).

### 2.7. In Vivo Pharmacokinetic Study

The pharmacokinetic study was conducted as an open-label, non-randomized, single dose comparative bioavailability study in 18 healthy adult volunteers under fasting conditions (20 subjects were enrolled in this study; however, 2 subjects did not complete this study due to intermittent illness). The single dose was administered along with 200 mL of water. The washout period was set to be at least seven days. Healthy adult male and non-pregnant, non-breast-feeding female volunteers, aged 18 to 55 years with body mass index 18.5 and 30 kg/m^2^ (inclusively), non- or ex-smokers were eligible to participate in this study. Subject eligibility was determined based on medical history, physical examination, 12-lead ECG, clinical chemistry, urinalysis, hematology, viral serology, blood pregnancy test for females, urinary drug screen, measurement of height and body mass (including calculation of BMI), vital signs measurement, alcohol breath test, and urinary cotinine test. Subjects were excluded if they had seated pulse rate below 50 or above 100 bpm, seated blood pressure below 90/60 or above 140/90 mmHg, documented hypersensitivity to omeprazole, substituted benzimidazoles or any excipients, positive screening HIV test results, hepatitis B surface antigen or hepatitis C virus test result, and history or presence of significant gastrointestinal, liver, kidney, or cardiovascular disease. Subjects with history of drug, tobacco, or alcohol abuse, or evidence of such abuse as indicated by the laboratory assays, were excluded from studies. Other key exclusion criteria were as follows: acute or chronic disease and/or clinical findings that might have influenced the study drug bioavailability, female subjects with positive blood/urine pregnancy test at screening or check-in, use of any prescription medicines less than 28 days before the first dosing (except of hormonal contraception or hormonal replacement therapy), and use of any over-the-counter (OTC) medication including vitamins, herbal medications, and food supplements less than 14 days before the first dosing. The consent form for participation was distributed to all participants and signed prior to the start of this study.

The omeprazole levels in plasma were determined by a validated liquid chromatography–tandem mass spectroscopic method. The HPLC/MS/MS system (Thermo Fisher Scientific, Waltham, MA, USA) was equipped with 1250 Transcend pumps, and a PAL HTS autosampler (CTC Analytics AG, Zwingen, Sweden) was employed. Chromatographic separation was achieved with gradient elution on a Luna C18(2) 100 Å (100 × 4.6 mm) analytical column fitted with a Luna C18(2) Mercury (20 × 4.0 mm) guard column from Phenomenex, (Torrance, CA, USA). The mobile phase consisted of MeOH and water at flow rate 0.6 mL/min. Monitoring of the analyte and respective internal standard was achieved using TSQ Quantiva tandem mass spectrometer (Thermo Fisher Scientific, Waltham, MA, USA) equipped with a heated electrospray ionization source (HESI) operating in the positive ionization mode. Quantitation was performed using selected reaction monitoring (SRM) for the following mass transitions: *m*/*z* 346 to 198 for omeprazole, and *m*/*z* 349 to 198 for d3-omeprazole. The linear dynamic range of the method in human plasma containing K2EDTA was from 10.0 to 3000.0 ng/mL. The concentrations were calculated using a linear regression model with weighted least squares (weight = 1/c, where c is the nominal concentration of the respective calibration sample).

Main pharmacokinetic parameters, such as maximum plasma concentration (C_max_), time to reach the maximum plasma concentration (t_max_), area under the plasma concentration–time curve from time zero to the time of the last quantifiable concentration (AUC_(0−t)_, measured by linear trapezoid method), AUC from time zero to infinity (AUC_(0−inf)_), and terminal elimination half-life (t_half_), were determined by non-compartmental methods using Phoenix WinNonlin, version 8.3 (Certara, Radnor, PA, USA). Statistical analysis was generated using SAS software, version 9.4 (SAS Institute Inc., Cary, NC, USA).

## 3. Results and Discussion

### 3.1. Chemical Stabilization of Omeprazole

The average liquid volume of the fasting stomach prior to ingestion of any water is reported to be 28 mL, with a range of 18–54 mL [25,26]. Omeprazole is known to be stabilized against acidic degradation when in a mixture with alkaline compounds. Hence, several such protectants were evaluated, testing their neutralizing activities in 54 mL of 0.1 M HCl. This is the maximum amount of gastric acid that would have to be neutralized to protect omeprazole against acid hydrolysis. Antacids screened in this study were bicarbonates (sodium, potassium), carbonates (calcium, magnesium), hydroxides (magnesium, aluminum), magnesium oxide, and meglumine.

The rates of pH increase and bulkiness of the material were used as the main parameters to select the stabilizing agent, as the aim of this study was to reduce the capsule size as much as possible, while still providing sufficient stabilization of omeprazole. The most ideal stabilizer would be the one with high bulk density (low volume), while also providing swift pH elevation. Based on these criteria, meglumine and MgCO_3_ were excluded from further evaluation early on due to bulkiness of these materials (high volume, due to low bulk density). Their use would result in large capsules that could eventually cause difficulties in swallowing, hence negatively affecting patient compliance. Among the other neutralizing agents, NaHCO_3_ and MgO showed superior pH increase and were selected for further studies. NaHCO_3_ exhibited the ability for rapid pH increase, while MgO, although demonstrating somewhat slower neutralization kinetics, achieved the highest pH levels.

As it was necessary to understand the release kinetics of omeprazole in the presence of acid-neutralizing agents, the next step was to perform in vitro dissolution measurements, including pH tracking in simulated gastric conditions. These measurements were expected to provide reliable information on the ability of MgO and NaHCO_3_ to protect omeprazole from degradation.

The mechanism of action of omeprazole protectants is based on simple neutralization, and can be described as follows:

HCl + NaHCO_3_ → NaCl + CO_2_+ H_2_O

2HCl + MgO → MgCl_2_ + H_2_O

Since one molar equivalent of NaHCO_3_ is required to neutralize one mol of HCl, 5.4 mmol of NaHCO_3_ would be required for 54 mL of 0.1 M acid, which is the maximum amount of gastric acid. However, if an equimolar amount of NaHCO_3_ was used, formation of CO_2_ and consequent acidification of solution could cause unwanted omeprazole degradation. NaHCO_3_ was, therefore, added in quantities greater than 5.4 mmol to ensure sufficient stabilization of omeprazole. The dissolution experiments, hence, involved varying amounts of NaHCO_3_, with two specific quantities introduced: 7.6 mmol and 13.1 mmol (the upper level corresponding to the quantity of NaHCO_3_ found in already marketed Zegerid). Only 13.1 mmol (matching the upper limit of the amount of NaHCO_3_) of MgO was used for this initial observation, with plans to test the lower quantities if the results of initial testing confirm suitability to use MgO. Amounts of stabilizing agents greater than 13.1 mmol were not considered due to bulkiness of the blend and the need for larger capsule size, which would be difficult to swallow.

Direct mixtures containing 20 mg of omeprazole and corresponding amounts of MgO or NaHCO3 were prepared, as per Table 1, and filled in the capsules. In vitro dissolution tests were conducted under simulated stomach conditions.

**Figure 3:**
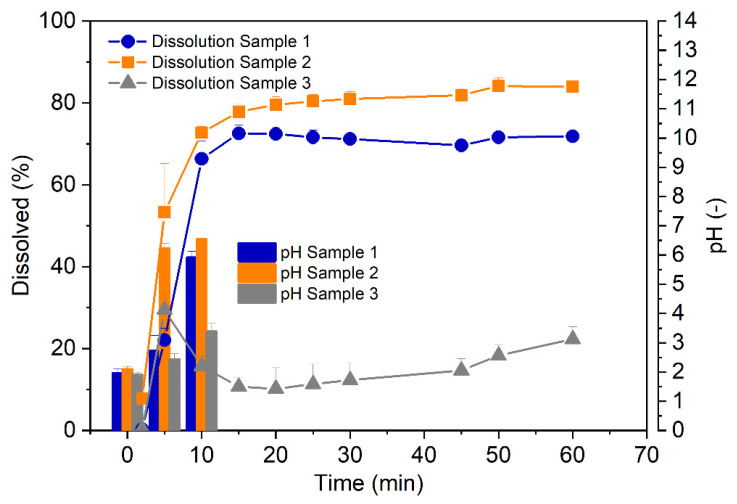
Cumulative dissolution profiles of omeprazole capsules containing as neutralizing agents either NaHCO_3_ (Samples 1–2) or MgO (Sample 3). Measurement of pH within the initial stage of the analyses (t = 10 min) showed that the sufficient pH increase by the MgO formulation (Sample 3) was not reached during that early stage, providing conditions for omeprazole degradation under acidic conditions.

Cumulative dissolution profiles of these formulations (prepared as per Table 1) are shown in Figure 3. It was demonstrated that the release of omeprazole occurred immediately, with measurable amounts of omeprazole already recorded at the first sampling point (5 min).

However, while the formulations of omeprazole with high (13.1 mmol) and low (7.6 mmol) amounts of NaHCO_3_ behaved similarly to each other, showing cumulatively increasing release of omeprazole, the amount of omeprazole released from the formulation containing MgO started to decline even in the initial stages of the experiment. Such a decline suggests that omeprazole undergoes chemical degradation, clearly demonstrating insufficient protection of omeprazole by MgO against acidic conditions (Figure 3).

While the dissolution results confirmed the protective and stabilizing effect of NaHCO_3_ in the formulation, the degradation of omeprazole observed in the formulation with MgO was unexpected. Moreover, at the end of the analysis of MgO-stabilized formulation, pH of the dissolution medium was significantly above the degradation threshold for omeprazole (approximately pH 10). This confirmed the initial minutes of acidic exposure to be critical for the degradation or stabilization of omeprazole. Indeed, measurement of the evolution of the pH in the medium during the initial minutes of the experiment revealed a direct correlation between the cumulative amount of omeprazole released and the magnitude of the initial pH increase (Figure 3). According to the existing literature data, if used without a protectant in acidic environment (pH < 5), at least half of the omeprazole degrades within the first ten minutes of the test [15]. The formulation containing MgO as an acid-neutralizing agent did not reach the threshold of pH 5 within those first 10 min (Figure 3), which resulted in the degradation of omeprazole. Additional research and a deep dive into the solid state of MgO would be needed to understand the reasoning behind the slower neutralization kinetics originating from MgO.

In summary, compared to MgO, the results of in vitro dissolution tests and pH measurements highlight the superior ability of NaHCO_3_ to rapidly neutralize gastric acid, effectively protecting omeprazole from degradation. The amount of 9.7 mmol of NaHCO_3_ was chosen to be used in the final formulation to account for an additional possible acidification during the neutralization reaction (formation of CO_2_) and individual variability in acid secretion.

Confirming the stability of the final formulation represented the next step of this study. Given the significant impact of moisture on product stability, the hygroscopicity of the formulation was further tested. Long-term chemical stability of the formulation was evaluated under standard room conditions, commonly used for storing pharmaceutical products.

### 3.2. Formulation Stability

#### 3.2.1. Hygroscopicity of Capsules

**Figure 4:**
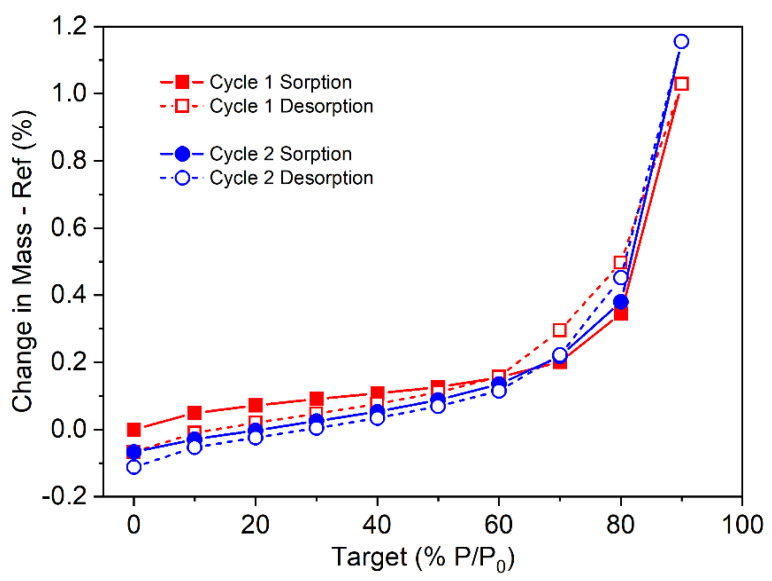
DVS analysis of omeprazole–NaHCO_3_ capsules indicating insignificant mass change under standard relative humidity (60%), confirming non-hygroscopicity of the formulation.

DVS analysis (Figure 4) performed on omeprazole–NaHCO_3_ capsules at room temperature (25 °C) showed low hygroscopicity under standard room conditions, with no significant moisture uptake observed. This finding suggests a minimal risk of chemical degradation of omeprazole–NaHCO_3_ caused by moisture.

#### 3.2.2. Chemical Stability of Capsules

Omeprazole–NaHCO_3_ capsules, packaged in PVDC/Al blisters, demonstrated stability at 3, 6, 9, and 12 months of storage, with no evidence of chemical degradation of either the drug or its protectant. The amounts of omeprazole and NaHCO_3_ remained within the required limits, as shown in Table 2. Purity tests using HPLC analysis of the capsule contents confirmed that impurity levels were within accepted thresholds. Additionally, the appearance of the capsule contents remained unchanged. The results confirmed chemical compatibility of the formulation components and stability of the formulation overall.

### 3.3. In Vivo Pharmacokinetic Study

Omeprazole–NaHCO_3_ hard gelatin capsules were used in a pharmacokinetic (PK) study conducted with healthy male and female volunteers under fasting conditions. The pharmacokinetic profile of the test formulation was compared to that of the reference formulation, delayed-release enteric-coated Losec 20 mg capsules. Key parameters, including maximum plasma concentration (C_max_), time to reach the maximum plasma concentration (t_max_), area under the plasma concentration–time curve from time zero to the time of the last quantifiable concentration (AUC_(0–t)_), AUC from time zero to infinity (AUC_(0–inf)_), and terminal elimination half-life (t_half_), were determined for both formulations (Table 3).

The results demonstrated that under fasting conditions, the 90% confidence intervals for the test (omeprazole–NaHCO_3_ capsules) to reference (Losec 20 mg enteric-coated capsules) and ratio of AUC_(0–t)_ of omeprazole fell within the standard bioequivalence acceptance range (80.00–125.00%), indicating equivalent bioavailability of both formulations. Given the chemical protection of omeprazole in the delayed-release (enteric-coated) formulation, the confirmed bioequivalence in AUC_(0–t)_ indirectly suggests the absence of chemically degraded omeprazole in the immediate-release formulation, which might otherwise occur in the acidic stomach environment. In other words, the in vivo results validated the in vitro findings, showing stable levels of omeprazole attributed to the rapid and efficient acid-neutralizing effect of sodium bicarbonate (pH 6 reached in less than 5 min).

**Figure 5:**
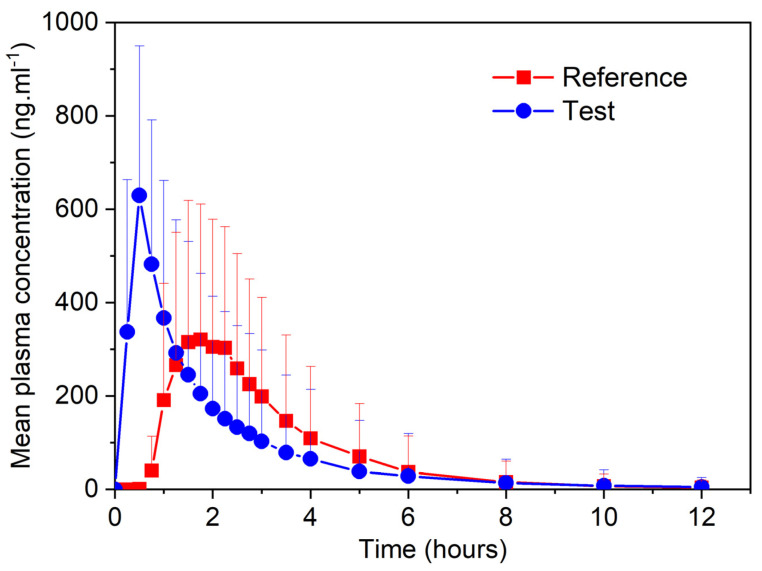
Mean plasma concentrations recorded during the pharmacokinetic study comparing omeprazole IR to enteric–coated pellets contained in Losec.

As seen in Figure 5, in comparison to the reference formulation, the t_max_ of the test formulation was noticeably shorter, being 30 min versus almost 2 h. The shorter t_max_ was expected due to the rapid release of omeprazole from the test formulation, which occurs immediately in the stomach, eliminating any barrier to fast omeprazole absorption. The first measured omeprazole plasma concentrations from the test capsules (at the 15 min sampling point) were comparable to the C_max_ of the delayed-release formulation. Moreover, at 30 min, which was the time when the immediate-release formulation had already reached t_max_, the absorption of the reference (delayed-release) formulation had just started, reaching its C_max_ at 1.75 h. Given that proton pump inhibition begins within 1 h of omeprazole administration [8], the faster absorption of the immediate-release formulation provides an earlier onset of proton pump inhibition and, subsequently, reduced acid secretion. Similar fast suppression of acid secretion was also observed with the immediate-release formulation of esomeprazole, which absorbed more rapidly compared to the delayed-release formulation [22].

Delayed absorption of the reference products was expected, as it consists of enteric-coated pellets that do not release omeprazole in the stomach (unlike the immediate-release formulation). The enteric coating physically protects omeprazole from exposure to acid (and consequent degradation), directing drug release to areas with higher pH. This mechanism results in a longer time needed to reach maximum plasma concentrations. On the contrary, the test formulation, due to chemical stabilization of omeprazole, does not differ from standard immediate release capsules, quickly releasing API already in the stomach.

The bioequivalence demonstrated by AUC_(0–t)_ and short t_max_ (30 min) of the test formulation indirectly confirms the acid-neutralizing effect of sodium bicarbonate and efficient protection of omeprazole from chemical degradation. Direct, rapid release of omeprazole within the neutralized gastric juice facilitated quick absorption from the small intestine.

However, it must be noted that the C_max_ of the test (immediate-release) formulation was higher than that of the reference (delayed-release) formulation. This was expected, as the test formulation releases the entire quantity of omeprazole within a short period of time, whereas the process is prolonged for enteric-coated pellets. This phenomenon needs to be considered in relation to safety and efficacy of the product. In this specific case, such supratherapeutic concentrations do not represent safety risks, as higher product strengths (40 mg formulations) are regularly used in clinical practice.

## 4. Conclusions

The aim of this study was to design an acid-neutralizing immediate-release formulation of omeprazole that prevents its chemical degradation, ensuring rapid release and absorption, while also providing technological insights in the design of such formulation. Since omeprazole undergoes chemical degradation in acidic pH environments, currently available formulations primarily rely on enteric coatings and further investigation of the stabilization mechanisms is desirable, on both API and formulation levels. In addition, incorporating more comprehensive in vivo studies or computational modeling could greatly aid in the selection and optimization of the formulation strategies. In this study, mechanisms of omeprazole stabilization were investigated without the use of enteric coatings. Since sodium bicarbonate showed superior rapidity in pH neutralization, a combination of omeprazole and NaHCO_3_ was formulated into immediate-release capsules. NaHCO_3_, by neutralizing the acid in the human gastrointestinal tract, prevents the degradation of omeprazole. The amount of used NaHCO_3_ was optimized to provide sufficient chemical stabilization of omeprazole while maintaining a sufficiently low concentration to support easy swallowing of the capsules. In vitro studies demonstrated the completion of acid neutralization in less than 5 min, enabling the fast release of omeprazole. In vivo studies further confirmed the rapid absorption of omeprazole and its availability for therapeutic effects. Plasma concentrations comparable to the C_max_ of the delayed-release formulation (achieved by Losec in 1.75 h) were reached within 15 min after omeprazole release from the immediate-release formulation. Maximum concentrations of omeprazole were achieved in just 30 min, whereas, at that time, the absorption of a standard delayed-release formulation only started. Since proton pump inhibition begins within 1 h of omeprazole administration, faster omeprazole absorption allows for earlier proton pump inhibition and an earlier onset in the reduction of gastric acid secretion. Maximum concentrations of omeprazole released from a delayed-release formulation were reached in almost 2 h. In other words, the synergy of omeprazole and NaHCO_3_ resulted in a stabilized all-in-one immediate-release formulation capable of achieving rapid acid neutralization, followed by rapid omeprazole release within the stomach, and rapid absorption from the small intestine.

## Figures and Tables

**Table 1:** Quantities of antacids added to 20 mg of omeprazole.

	Sample 1	Sample 2	Sample 3
NaHCO_3_ content (mmol/mg per capsule)	7.6/638.4	13.1/1100	
MgO content (mmol/mg per capsule)			13.1/527.8

**Table 2:** Chemical stability testing of omeprazole–NaHCO_3_ capsules after 12 months.

Test	Limit	Initial	3 Months	6 Months	9 Months	12 Months
Appearance of the capsule content	White to off-white powder	Complies	Complies	Complies	Complies	Complies
Average mass of 1 capsule content	0.817–0.904 g	0.861 g	0.861 g	0.862 g	0.863 g	0.861 g
Assay of omeprazole	95.0–105.0%	99.5%	100.8%	98.2%	100.8%	100.1%
Assay of NaHCO_3_	90–110%	101.0%	100.7%	100.8%	101.0%	100.9%
Total impurities	<2.00%	<0.05%	<0.05%	<0.05%	<0.05%	0.08%

**Table 3:** Pharmacokinetic parameters and geometric least-square means ratios (90% CI) of omeprazole following a single dose of test and reference formulations.

PK-Metric	Mean ± SD		GMR (90% CI)
	Test (n = 18)	Reference (n = 18)	Test vs. Reference
AUC_(0–t)_ [ng.h/mL]	1031.7 ± 1368.1	950.7 ± 1092.1	104.09 (96.71–112.03)
AUC_(0– inf)_ [ng.h/mL]	1068.7 ± 1443.1	982.3 ± 1148.0	104.32 (97.04–112.16)
C_max_ [ng/mL]	658.1 ± 316.4	446.4 ± 261.1	153.28 (135.76–173.08)
t_max_^a^ [h]	0.50 (0.25–0.75)	1.75 (1.00–5.00)	N/A
t_half_ [h]	0.99 ± 0.49	0.96 ± 0.49	N/A

^a^ Median (range); abbreviations: CI—confidence interval, GMR—geometric mean ratio, SD—standard deviation.

## Data Availability

The original contributions presented in this study are included in the article. Further inquiries can be directed to the corresponding author.
